# Effects of exercise training on behavior and brain function after high dose isoproterenol-induced cardiac damage

**DOI:** 10.1038/s41598-021-03107-z

**Published:** 2021-12-08

**Authors:** Kata Tóth, Tamás Oroszi, Eddy A. van der Zee, Csaba Nyakas, Regien G. Schoemaker

**Affiliations:** 1 Department of Neurobiology, GELIFES, University of Groningen, Nijenborgh 7, 9747 AG Groningen, The Netherlands; 2 Research Center for Molecular Exercise Science, University of Physical Education, Budapest, Hungary; 3 Behavioral Physiology Research Laboratory, Health Science Faculty, Semmelweis University, Budapest, Hungary; 4 University Medical Center Groningen, Groningen, The Netherlands

**Keywords:** Neuroscience, Systems biology, Medical research

## Abstract

Acute sympathetic stress can result in cardiac fibrosis, but may also lead to mental dysfunction. Exercise training after isoproterenol (ISO)-induced acute sympathetic stress was investigated regarding cardiac damage, neuroinflammation, brain function and behavior. Male Wistar rats (12 months) received ISO or saline. One week later, treadmill running or control handling (sedentary) started. After 4 weeks, cognitive- and exploratory behavior were evaluated, and heart and brain tissues were analyzed regarding cardiac damage, hippocampal neuroinflammation and neuronal function. ISO did not affect cognitive performance nor hippocampal function. However, ISO reduced anxiety, coinciding with locally reduced microglia (processes) size in the hippocampus. Exercise in ISO rats reversed anxiety, did not affect microglia morphology, but increased brain function. Thus, exercise after ISO did not affect cardiac damage, cognition or hippocampal neuroinflammation, but normalized anxiety. Increased localized BDNF expression may indicate improved brain function.

## Introduction

Acute sympathetic stress is characterized by overactivation of beta-adrenergic receptors, resulting in cardiac fibrosis and ultimately cardiac dysfunction^1^. Acute administration of high dose of beta-receptor agonist isoproterenol (ISO) is commonly used to mimic this clinical condition, as extensively reviewed by Nichtova et al.^2^. Acute ISO administration produced tachycardia, associated with relative ischemia due to imbalance between increased myocardial oxygen demand and reduced coronary blood supply in the heart. This evoked inflammation and increased cytokine production resulting in cardiac fibrosis^1,3^, that over a period of weeks developed into left ventricular hypertrophy and dilatation, and ultimately heart failure^2^. Although effects of potential treatment on cardiovascular aspects are widely investigated in the ISO model^1,4–6^, effect on brain and behavior are only sparsely studied^7–9^. Effects in the brain included increased apoptosis and reduced mitochondrial function after ISO though^8^. Exploratory behavior 6 days after ISO (+pituitrin) could be improved by Corvitin and 2-Oxoglutarate^7^, ISO-induced depressive behavior was declined by the Chinese medicine Kai-Xin-San^9^, while cognition, tested 2–8 days after ISO, could be improved by sodium thiosulfate^8^.

Exercise training is generally acknowledged for having positive effects on physical as well as mental conditions^10^. Exercise in the ISO model inhibited cardiac fibrosis in an adenosine monophosphate-activated protein kinase-dependent way^4^. Exercise training before ISO attenuated the acute β-adrenergic overactivation and inhibited cardiac fibrosis^1^. However, in most exercise intervention studies, the exercise started weeks before ISO, suggesting prevention rather than treatment of consequences of ISO. As Alemasi et al.^1^ already indicated, acute stress is often caused by unexpected events and is difficult to predict. Therefore, it seems worthwhile to explore interventions that could be applied after ISO had exerted its effect on the heart, and cardiac fibrosis was eminent. Exercise is well known for its anti-inflammatory effects^11^. Evidence is accumulating for an association between mental dysfunction and neuroinflammation^12^. Although exercise was shown to prevented the upregulation of 18 cytokines^1^, in none of the above-mentioned behavioral studies in the ISO model^7–9^, effects on (neuro)inflammation were studied.

Aim of the present study was to investigate effects of exercise training, starting after cardiac damage induced by high dose of ISO has been established, with focus on the brain. For that, effects on neuroinflammation, neuronal function and behavior were studied.

## Material and methods

### Animals and experimental protocols

The study has been separated into two experiments. First, to validate the model of ISO in our hands, the short-term effects of ISO on mortality, cardiac function and damage, and neuroinflammation were studied in young rats. Because of availability of ECHO-cardiography equipment for rats necessary to measure cardiac function, this part of the study was performed in Groningen. For that, male 3 months old Wistar rats (Harlan), housed 2–3 per cage with food and tap water available at lib, were used. All methods were performed in accordance with the ARRIVE guidelines. All experiments were performed in accordance with relevant guidelines and regulations/legislations. Experimental animals and procedures were approved by the local animal committee of the University Groningen, the Netherlands. Rats were randomized to receive either ISO (n = 13) or saline (n = 3) treatment. One week later, rats were anesthetized for cardiac function assessment. Subsequently, rats were sacrificed and heart- and brain tissues were dissected and processed for (immuno)histochemistry.

The actual study, long term effects of physical training after ISO, was performed in Budapest, for the local availability of adult rats, and exercise equipment (treadmill). For that, 12 months old Wistar rats (breeding colony of University of Physical Education, Budapest, Hungary) were used. All methods were performed in accordance with the ARRIVE guidelines. All experiments were performed in accordance with relevant guidelines and regulations/legislations. Experimental animals and procedures were approved by the local animal committee of the University of Physical Education, Budapest, Hungary. Rats were randomized to receive either ISO (n = 35) or saline treatment (n = 20). One week later, rats were randomized to physical exercise by treadmill running, or were handled but remained sedentary for 5 weeks. During the sixth week following the injections, behavioral testing was performed, and rats were subsequently sacrificed. Heart and brain tissues were dissected and processed for (immuno)histochemistry.

### ISO treatment

Animals were randomly assigned to the two experimental interventions. The experimental group received 70 mg/kg ISO (Sigma Aldrich) dissolved in 1 ml/kg saline via intraperitoneal injections, control rats received 1 ml/kg saline. Twenty-four hours later, this treatment was repeated. This ISO protocol was based on the study of Ravindran et al.^8^, using 80 mg/kg in male Wistar rats of 250–300 g. As effects of ISO are more extensive in aged compared to young rats^13^, a slightly lower dose of ISO (70 mg/kg) was used in order to limit mortality.

### Cardiac function measurement

Cardiac function was estimated with transthoracic echocardiography (Sonos 5500, Philips, The Netherlands). For that, isoflurane anesthetized (± 2% in air/oxygen = 2/1) rats were placed in supine position on a heating pad to maintain body temperature at 37 °C. Standard two-dimensional and M-mode long- and short-axis images at the midpapillary level were acquired using a 12–13 MHz transducer. Left ventricular dimensions were obtained and calculated into fractional shortening and ejection fraction as measures for left ventricular function.

### Exercise

Exercise training (running) was performed on a six-lane rat treadmill (Tartonik Elektronika, Italy) with individual lanes of 12 × 54 × 13 cm. The training program lasted for 5 weeks, 5 times per week on each weekday, and was based on the study of Azamian et al.^14^. On the first week of the training program rats were habituated to running: on the first day 10 min of running with a maximal speed of 10 m/min, which was gradually raised to 30 min and maximal speed of 18 m/min by the fifth day. For the following 4 weeks each running session lasted 30 min: starting with a 5-min warm up to reach the desired speed. Running at a speed of 18 m/min was considered a moderate intensity of approximately 65% of VO_2_max (pilot study).

### Behavior

Different aspects of behaviour were assessed. Effects on anxiety were assessed by exploratory behavior in an open field (OF) test. Cognition was measured as short-term memory in the novel object recognition test (NOR) and the novel location recognition test (NLR). All tests were recorded with a digital video camera and stored on a memory card. Tests were carried during at the end of the 5 week’s intervention, and encompassed 10 days.

#### Open field exploration

Rats were placed in a round shaped arena and were given 5 min to freely explore it, while behavior was recorded. The arena was divided into wall and center areas, delineated by concentric circles. Time spent in the areas were measured from the recordings by Eline (University of Groningen), regarding time spent in center and wall areas. Percentage of time spent in the area along the wall was taken as a measure for anxiety/depressive-like behavior. After removal of animals, the arena was cleaned with 70% ethanol and paper tissue.

#### Novel object and novel location recognition

The two memory tests were combined in one protocol^15^. The rat was placed in a box shaped arena and was let 3 min to get accustomed to the settings, then two identical objects were placed into the arena. After again 3 min the objects were removed and cleaned, and 1 min later, one familiar and one novel object were placed back on the same locations. Again, after 3 min objects were removed, cleaned, and after 1 min placed back but one of the objects was moved to a new location. Test was ended 3 min after exploration of the last setting. Arena and objects were cleaned with 70% ethanol after each animal. Time spent exploring the objects was measured (Eline). Preference for the novel object (NOR) or novel location (NLR) was calculated as percentage time spent exploring the novel/relocated object divided by time spent exploring both objects.

### (Immuno)histochemistry

#### Cardiac collagen

Since ISO was anticipated to cause focal myocardial infarcts, percentage collagen was used to measure cardiac damage. For that, 20 µm thick transverse slices at mid-ventricular and apex level of the heart were stained with Sirius red (Sigma, Aldrich) and fast green as counter staining^16^. Colour pictures were taken. Image analysis (Image Pro plus, USA) was used to measure the collagen positive (red) area and was expressed as percentage of total left ventricular tissue area.

#### Neuroinflammation

Since microglia are regarded the immune cells of the brain, and change shape when activated, microglia morphology was used to measure microglia activity^17^. We focussed on the hippocampus as memory processes and anxiety share involvement of this brain structure^18^. In IBA-1(Wako, USA) stained brain slices (20 µm thick), different hippocampal areas, Cornu Ammonis (CA)1 and Hilus^15^ were photographed (200×), and microglia were analysed, according to altered morphology, including density, coverage, cell size, cell body size and processes size. Microglia cell body to cell size ratio was used as a measure for microglia activity; neuroinflammation^17^.

#### Brain function

For brain function, brain slices were stained with Brain Derived Neurotrophic Factor (BDNF) antibody (Alomone Labs, Israel). In the different areas of the dorsal hippocampus, CA1, CA3, Dendate Gyrus and Hilus, BDNF expression was obtained as corrected optical density (Image-J) compared to an underlying reference area^15^.

### Data analyses

The study has been reported in accordance with ARRIVE guidelines. All reports were performed in accordance with relevant guidelines and regulations/legislations. Data are presented as mean and standard error of mean (SEM), unless indicated otherwise. Results outside twice the standard deviation of its group were considered outliers and were excluded before analyses (maximally 1–2 per experimental group). Data of the first study, to establish the ISO model, were compared with a student’s T test for independent samples, for ISO versus saline treatment. Data of the actual study, effects of long-term exercise in the ISO model, were compared using two-way analysis of variance (ANOVA) with least square difference (LSD) post-hoc test, with saline/ISO and sedentary/runner as factors. Association between selected parameters were measured with Pearson linear correlation. For the Novel Object /Novel Location recognition test, outcomes were also tested against change level (= 50%), using a single sample t test. A p value of < 0.05 was considered statistically significant and presented as *. Potentially relevant tendencies (p < 0.1) were mentioned as well.

### Ethical approval of animal studies

The animal experiments were approved by the animal committee of the University Groningen, the Netherlands and the animal committee of the University of Physical Education, Budapest, Hungary.

## Results

### The ISO model

**Figure 1:**
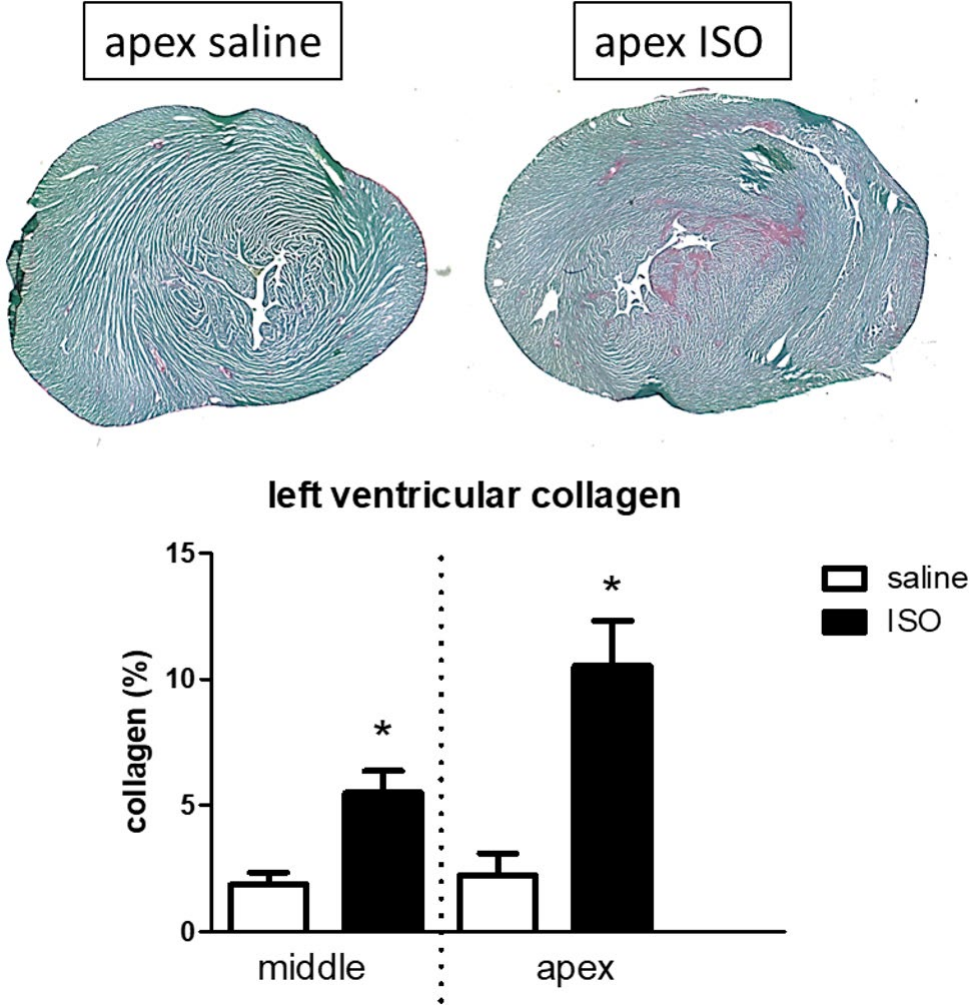
Photographs of Sirius Red/Fast green stained apical heart slices from rats treated with saline or isoproterenol (ISO), showing more collagen (red) in ISO treated hearts. Lower panel shows actual measurement of percentage collagen 1 week after ISO or saline treatment. *Significant effect of ISO.

Before studying the effects of exercise in the ISO model, we wanted to validate the model of ISO in our hands, regarding mortality, cardiac function and damage, and neuroinflammation, and to provide information about the conditions 1 weeks after ISO, as the start point of the exercise intervention. Although rats exhibit symptoms described by Wexler and Kittinger^19^, including prostrate and stuporous behavior, with irregular breathing, they recovered well and mortality was 14% in these young rats. One week after the injections, heart and lung weights were not affected by ISO (Table 1), and no decline in cardiac function was observed (Table 2). However, cardiac collagen percentage, representing damage, was significantly higher after ISO, both at the mid-ventricular level and at the apex (Fig. 1). Absence of increased tissue area in microscopical slices supported the lack of increased heart weight. Apart from a significantly decreased relative brain weight, no effects of ISO were observed on organ weights.

**Table 1:** Body- and organ weights, 1 week after isoproterenol (ISO) or saline treatment.

	Saline N = 3	ISO N = 11
Body weight (g)	360 ± 6	391 ± 7*
Heart weight (% of body weight)	0.34 ± 0.02	0.34 ± 0.01
Lung weight (% of body weight)	0.40 ± 0.02	0.39 ± 0.01
Liver weight (% of body weight)	3.54 ± 0.12	3.34 ± 0.31
Spleen weight (% of body weight)	0.22 ± 0.01	0.22 ± 0.02
Left adrenal gland (% of body weight)	0.007 ± 0.001	0.006 ± 0.0004
Brain weight (% of body weight)	0.56 ± 0.01	0.52 ± 0.01*

*Significant effect of ISO.

**Table 2:** Echocardiograpically obtained parameters of cardiac dimensions and cardiac function, 1 week after isoproterenol (ISO) or saline treatment.

	Saline N = 3	ISO N = 11
Heart rate (beats/min)	396 ± 21	379 ± 9
Left ventricular end-diastolic diameter (mm)	72 ± 2	77 ± 2
Left ventricular end-systolic diameter (mm)	31 ± 3	35 ± 2
Fractional shortening (%)	57 ± 3	55 ± 2
Left ventricular ejection fraction (%)	89 ± 1	90 ± 1

*Significant effect of ISO.

Neuroinflammation in the hippocampus, represented by microglia activity in the CA1 and hilus, was not significantly affected 1 week after ISO treatment (CA1 area: saline 5.3 ± 0.5%, ISO 5.3 ± 0.3%; Hilus: saline 14.7 ± 1.9%, ISO 11.3 ± 0.7%). Neither did any of the other microglia morphology parameters changed, including density, coverage, cell size, cell body size and processes size.

### Effects of ISO and exercise

#### Mortality

From 35 male rats injected with ISO, 15 rats died within the first days. No mortality was observed in 20 saline treated rats, resulting in 10 rats in each of the experimental groups; saline sedentary; saline runner; ISO sedentary; ISO runner.

#### Effects on the heart

General characteristics of the experimental groups are summarized in Table 3. Two-way ANOVA revealed a significant effect of ISO versus saline for heart weight, but no effect of running, nor interaction between saline/ISO and sedentary/runner.

**Table 3:** General characteristics of the experimental groups (n = 10 each), before and 6 weeks after isoproterenol (ISO) or saline treatment, with or without exercise (runner).

	Saline sedentary N = 10	Saline runner N = 10	ISO sedentary N = 10	ISO runner N = 10
Body weight start (g)	448 ± 19	442 ± 11	430 ± 12	426 ± 8
Body weight end (g)	438 ± 22	437 ± 15	430 ± 13	420 ± 7
Heart weight (% of body weight)	0.30 ± 0.01	0.28 ± 0.01	0.32 ± 0.01	0.32 ± 0.01*
Brain weight (% of body weight)	0.50 ± 0.02	0.49 ± 0.02	0.49 ± 0.01	0.49 ± 0.01

*Post-hoc analyses revealed a significant effect of ISO runner versus saline runner.

**Figure 2:**
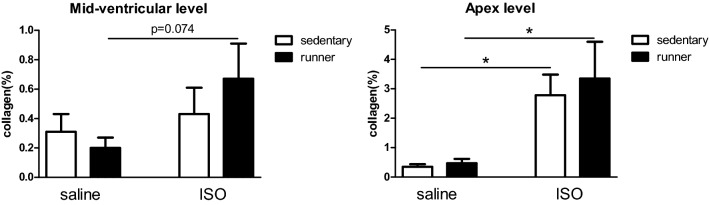
Collagen positive area in the left ventricle of the heart, at mid ventricular level and apex, in saline and isoproterenol (ISO) treated rats, and effects of exercise (runner) versus sedentary conditions. *Significant difference between saline and ISO.

Cardiac collagen levels, as measure for ISO-induced focal infarcts, were significantly increased at the apex of the heart (Fig. 2). Two-way ANOVA revealed a significant effect of ISO, which appear most pronounced in runners. No effect of sedentary versus running, nor interaction effects were observed. Apical collagen, but not mid-level collagen, was significantly correlated to heart weight (r = 0.55, p < 0.000).

#### Effects on the brain

**Figure 3:**
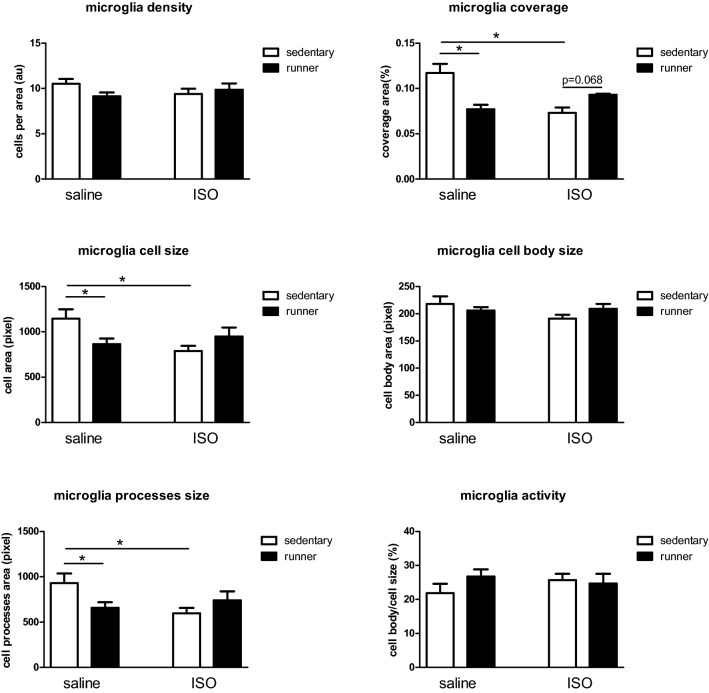
Microglia morphology parameters obtained from the Hilus of the hippocampus in saline and isoproterenol (ISO) treated rats, and effects of exercise (runner) rats versus sedentary conditions. *ISO* isoproterenol treatment; *significant difference between indicated groups (n = 9–10 per group).

Neuroinflammation was obtained from morphologic changes in microglia in the CA1 and Hilus area of the hippocampus. No significant differences were observed in microglia parameters in the CA1 area (Table 4). In the Hilus (see Fig. 3), ISO decreased the size of the microglia by decreasing the area covered by processes. This did not result in increased microglia activity measures, although cell body size seemed unaffected. The reduced microglia size was not compensated for by increased density, resulting in reduced coverage (Fig. 3). No correlations were observed between open field behavior and Hilus microglia parameters. Running in saline treated rats had similar effects on microglia morphology as had ISO. However, running in isoproterenol-treated rats, may partly reverse the ISO-induced declined microglia coverage.

**Table 4:** Parameters of microglia morphology in the CA1 area of the hippocampus in saline and isoproterenol (ISO) treated male rats, and effects of exercise (runner) rats versus sedentary conditions.

	Saline sedentary N = 10	Saline runner N = 10	ISO sedentary N = 10	ISO runner N = 10
Density (# cells/area)	4.86 ± 0.21	4.79 ± 0.10	4.74 ± 0.19	4.86 ± 0.21
Coverage (% area)	9.9 ± 0.6	9.9 ± 0.7	8.4 ± 0.4	10.6 ± 0.9
Cell size (pixel)	2102 ± 151	2146 ± 187	1825 ± 71	2268 ± 255
Cell body size (pixel)	182 ± 7	177 ± 5	181 ± 10	184 ± 8
Processes size (pixel)	1919 ± 154	1969 ± 188	1644 ± 73	2085 ± 255
Activity (cell body/cell size; %)	9.6 ± 0.9	9.0 ± 0.7	10.7 ± 0.8	9.3 ± 1.0

**Figure 4:**
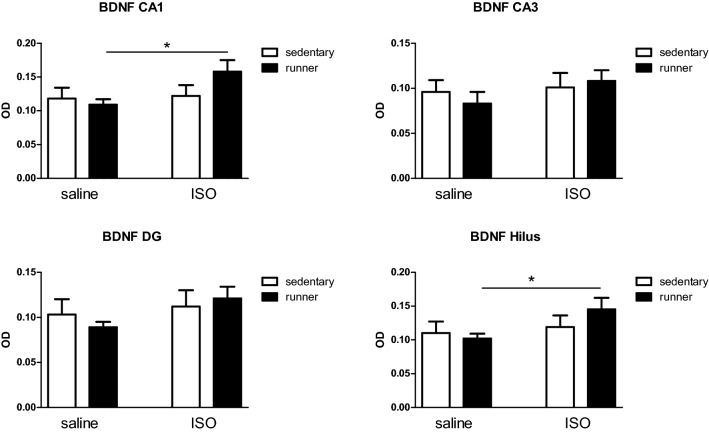
Brain derived neurotrophic factor (BDNF) expression in the different regions of the hippocampus in saline and isoproterenol (ISO) treated rats, and effects of exercise (runner) rats versus sedentary conditions. *Significant difference between saline runners and ISO runners.

Since the hippocampus is mostly involved in learning and memory, function of the brain was estimated by Brain Derived Neurotrophic Factor (BDNF) expression in the hippocampus. In sedentary animals, no effects of ISO were observed in any of the areas. Running exercise was not affecting BDNF in saline treated rats. However, in all areas BDNF expression in ISO treated rats with exercise was slightly higher than in saline treated rats, reaching statistical significance in the CA1 and Hilus areas (Fig. 4).

#### Effects on behavior

**Figure 5:**
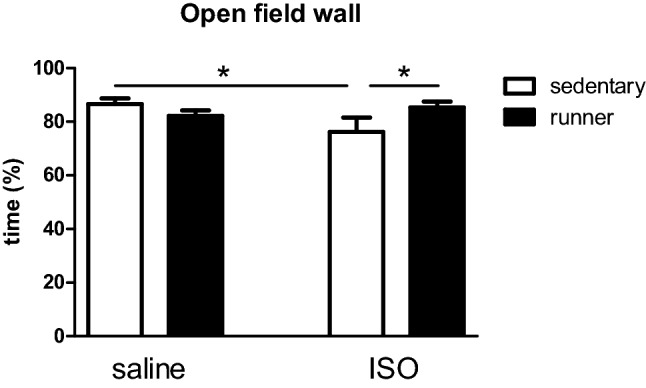
Time spent in the wall area of the open field in saline and isoproterenol (ISO) treated rat, and effects of exercise (runner) versus sedentary conditions. *Significant difference between indicated groups.

General exploratory behavior was obtained from the open field test. Anxiety/depression levels were considered as more time spent at the wall areas rather than in the center of the open field. Two-way ANOVA revealed no significant effect of either saline/ISO or sedentary/running alone, but a significant interaction between both factors, resulting in post-hoc analysis of significantly decreased time spent at the wall area after ISO, which was reversed by running (Fig. 5).

**Figure 6:**
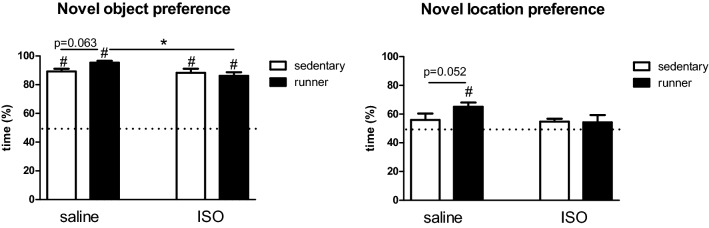
Cognitive effects of isoproterenol (ISO) versus saline treatment, and the effects of running versus sedentary conditions. *Significant difference between indicated groups; ^#^significantly different from chance level (= 50%; dashed line).

Cognitive behavior was tested in the Novel Object Recognition (NOR) and the Novel Location Recognition (NLR) tests. Figure 6 shows the results of these tests. In the NOR test, all groups performed significantly above chance level (= 50%). Running may slightly improve performance in saline treated rats, but not in ISO treated rats. Although in the NLR test only saline runners performed above chance level, similar effects as seen in the NOR test were observed.

## Discussion

### General

Aim of the present study was to investigate the effects of 5 weeks exercise training on hippocampal neuroinflammation, neuronal function and behavior after established ISO-induced cardiac damage. At the start of exercise training, 1 week after ISO, cardiac damage seemed eminent, without declined cardiac function or neuroinflammation (microglia activity). Although long-term cardiac damage after ISO appeared substantial, it did not induce cognitive impairment nor associated changes in microglia or BDNF expression in hippocampal CA1. However, long-term effects of ISO suggested altered microglia morphology in the Hilus and reduced anxiety in the OF. Five weeks of running reversed the reduced anxiety, but did not affect cognition or neuroinflammation in ISO rats. However, running treatment after ISO-induced cardiac damage may increase brain function, measured by BDNF expression.

### The model

Acute sympathetic stress is associated with overactivation of beta-adrenergic receptors, leading to cardiac damage and ultimately cardiac dysfunction and heart failure^1,20,21^. However, acute stress is often caused by unexpected events and therefore difficult to predict^1^. Acute administration of high dose of the beta-receptor agonist isoproterenol (ISO) is commonly used to mimic this clinical condition^21^. Although interventions in this model were mostly aimed at prevention of the cardiac damage, regarding the unpredictable character of the clinical condition, it seemed worthwhile to explore interventions that could be applied after ISO had exerted its effect on the heart, and cardiac fibrosis was eminent. Interventions that target consequences of cardiac damage were mostly investigated in the more labor-intensive and technical skills requiring model of coronary artery ligation^22^. Moreover, our previous study^16^ indicated a mixed effect of the surgical procedure for coronary artery ligation and the effect of the subsequent introduced cardiac damage, on cognition and (neuro)inflammation. The model of ISO-induced cardiac damage circumvented these additional surgery-induced effects.

### Effects of ISO

ISO, administered twice with 24 h in between, evoked inflammation and cytokine production and cardiac fibrosis^1^, that over a period of weeks developed into left ventricular hypertrophy and dilatation, and ultimately heart failure^2^. Effects of ISO appeared age dependent^23,24^. Accordingly, in the present study mortality in the adult (12 months old) animals was substantially higher than in the young (3 months old) rats. The presence of cardiac damage and scar formation 1 week after ISO administration in young rats indeed implicated activation of the innate immune response necessary for wound healing. This is in accordance with the upregulation of 18 cytokines, 3 days after ISO, leading to fibrosis 7 days after ISO^1^. Moreover, the cardiac inflammatory response was reflected in the circulation by increased levels of key proinflammatory cytokines, such as IL6 and TNFα^25^. Increased inflammatory mediators (specifically TNFα) in the circulation, associated with cardiac damage, can be reflected in the brain as neuroinflammation, associated with depressive-like behavior^26^, by mechanisms that include direct passage of cytokines through the blood brain barrier by leakage or active transport, as well as peripheral afferent nerve stimulation^27^. However, 1 week after ISO, microglia morphology was not altered, indicating no signs of neuroinflammation at the time our exercise intervention started. Also, cardiac function had not declined at 1 week yet. Accordingly, up to 1 week after injections, blood pressure, cardiac output and work were in the normal range under basal conditions, but maximum cardiac output and work were well below normal^20^. Normal heart weight and tissue coverage (microscopical sections) suggested cardiac myocyte hypertrophy to compensate for the loss of viable myocardium; compensated cardiac hypertrophy. Absence of increased lung weight supported absence of overt heart failure^20^. Six weeks after ISO in 12 months old rats, cardiac fibrosis was still present, though less pronounced than in 3 months old rats 1 week after ISO. This would be in agreement with the old study of Beznak et al.^20^; during the first few weeks following ISO injection, histological repair took place, and the cardiovascular parameters measured were not different from those found in naive rats^20^.

Although direct effects of beta-adrenergic stimulation on behavior have been studied; long-term effects of this acute beta stimulation on neuroinflammation and behavior are less well known^7^. Still, ISO seemed to reduce exploratory behavior and the autonomic emotional state^7^, reduce depressive-like behavior^9^ and induced cognitive decline^8^. In the present study, no cognitive effects of ISO were observed. Since the used cognitive tests were mainly aimed at hippocampal function, accordingly microglia morphology was analyzed in this brain structure. In accordance with the lack of cognitive effects, no microglia activity was observed in the CA1 area of the hippocampus, neither was brain function, measured by hippocampal BDNF expression, affected by ISO. Surprisingly, ISO seemed to have reduced anxiety, as measured by less time spent at the wall area in the open field. This would be in contrast to our previous findings in coronary artery ligation rats^28^. The effect on open field behavior coincided with altered microglia morphology in the hippocampal Hilus area. This would be in agreement with our study in coronary artery ischemia–reperfusion induced cardiac damage, showing microglia activation in the Hilus, but not in the CA1 area^16^. However, in the previously mentioned study declined cognitive performance in the NLR test was observed, which was not seen in the current investigation. An explanation could be that in the present study at 12 months of age, even control rats did not recognize the relocated object. Therefore, the effects of ISO to mimic the consequences of acute sympathetic stress-induced cardiac damage, seemed in general agreement with literature, and showed similarities with the consequences of myocardial infarction-induced by coronary artery ligation in our hands.

### Effects of exercise

Exercise training is generally acknowledged for having positive effect on physical as well as mental conditions. Moreover, exercise is known to have anti-inflammatory effects^11^. The potentially anti-inflammatory effect of exercise could counteract (neuro)inflammation and thereby improve cognition and mood^29^. ISO induced an inflammatory response for healing and focal cardiac scar formation. Exercise started 1 week after ISO did not interfere with cardiac fibrosis. However, exercise starting 2 days after ISO, that is within the inflammation phase^1^, exaggerated cardiac fibrosis^14^. This discrepancy may be explained by the time course of inflammation and scar formation, progressing into completion at 1 week. Six weeks of running starting before ISO reduced cytokines expression 3 days after ISO and limited cardiac fibrosis^1^. Moreover, 14 weeks of exercise prior to ISO did reduce myocardial damage, but this seemed not mediated by indomethasin-induced reduction of prostacyclin^30^. Above results may indicate that exercise indeed would be able to reduce inflammation and cardiac damage after ISO, but effects highly depend on timing. Moreover, the only study we are aware of where exercise started after ISO, reported increased rather than reduced cardiac fibrosis^14^.

Behavioral effects of exercise are only sparsely investigated in the ISO model. Exercise reversed the ISO effect in open field behavior by normalizing anxiety. Exercise tended to improve cognitive performance in saline treated rats, but not in ISO rats. In fact, only saline treated rats that had exercise training performed above chance level in the NLR test. The positive effects of exercise on depression and cognition have been related to increased levels of neurotrophic factors, elevated expression of anti-inflammatory cytokines, and reduced levels of pro-inflammatory cytokines and activated microglia (reviewed by Svensson et al.^29^). Exercise-induced stimulation of brain BDNF is well-known^31^. Accordingly, in the present study, exercise increased BDNF expression in both the CA1 as well as hilus area of the hippocampus, but only in ISO-treated rats. Morphological changes indicated a reduction of microglia size, mainly due to lower processes area after ISO. Although not reflected in increased microglia activity, measured as cell body size/cell size^17^, a lower processes area would indicate de-ramification of the microglia, which is usually associated with activation^32^. Exercise may partly reverse this microglia activation in ISO rats. Although exercise is often performed as prevention rather than reversal of effects of ISO^1,4^, or coronary ligation-induced MI^33^, effects are generally going in the same direction as seen in the present study. Interestingly, exercise training is reported to affect glycogen metabolism in skeletal muscles^34^, and in the brain glycogen metabolism was associated with neuroplasticity^35^. The Hilus appeared to be the most glycogen-rich subregion of the hippocampus^36^. Accordingly, in the present study, the most pronounced effects of exercise in the hippocampus were observed in the Hilus area. Although the hippocampus was our area of interest based on literature, it cannot be excluded that other brain regions show significant effects of exercise as well.

### Limitations

Each study has its limitations. The first part of the study was aimed at developing the ISO model in our lab. For that, young male Wistar rats were used. Based on the outcome of established cardiac damage 1 week after ISO treatment, in the subsequent study, the intervention of exercise training was started from 1 week onwards. This latter study was performed in 12 months old male Wistars as adult rats. Since the effects of ISO seemed age dependent, we chose for adult rats, to mimic the population of patients experiencing acute sympathetic stress. Indeed, mortality in these adult rats appeared higher than in the young rats. However, whereas the young rats were studied in Groningen and the adult rats in Budapest, differences in facility condition may have contributed as well.

Moreover, timing of the exercise intervention, from 1 to 6 weeks after ISO treatment, was chosen carefully regarding the aim of exercise intervention; treatment rather than prevention of an unpredictable event. However, it could be that the process of inflammation-neuroinflammation that we aimed to interfere with, was merely complete at 1 week after ISO, hence limiting effects of our exercise intervention. Other starting time as well as duration could have provided different results.

No blood samples have been collected from these rats to evaluate levels of circulating inflammatory markers, which could have helped to elucidate on the underlying mechanisms.

Finally, since no echocardiography equipment was available in the lab in Budapest, effects of exercise training on cardiac function could not be obtained. Although cardiac damage, as percentage fibrosis was not altered by exercise, the increase in heart weight in the ISO + exercise group could have pointed at changes in cardiac function.

## Conclusion

Aim of the present study was to investigate the effects of 5 weeks exercise training on neuroinflammation, neuronal function and behavior after established ISO-induced cardiac damage. Although cardiac damage after ISO appeared substantial, it did not induce long-term cognitive impairment nor associated changes in the hippocampal CA1 area. Accordingly, exercise training did not affect these aspects. Surprisingly, ISO seemed to reduce anxiety, which was reversed by exercise. ISO-induced microglia activation in the Hilus was not significantly affected by exercise, but exercise in ISO-treated rats showed increased brain function. In conclusion, 5 weeks of exercise, starting 1 week after ISO treatment, did not affect ISO-associated cardiac damage, cognition or neuroinflammation, but normalized the reduced anxiety shown in the open field. Increased localized BDNF expression may indicate improved brain function. Additional behavioral tests, and measurement of circulating inflammatory markers in a follow-up study may further elucidate on the underlying mechanisms.

## Abbreviations

BDNF: Brain-derived neurotrophic factor
CA: Cornu Ammonis
EF: Ejection fraction
ISO: Isoproterenol
Iba-1: Ionized-binding adaptor protein 1
LSD: Least square difference
NLR: Novel location recognition test
NOR: Novel object recognition test
OF: Open field
SEM: Standard error of the mean
VO_2_max: Maximal oxygen uptake capacity

## References

[1] Alemasi A, Cao N, An X, Wu J, Gu H, Yu H, Song Y, Wang H, Zhang Y, Xiao H, Gao W (2019). Exercise attenuates acute beta-adrenergic overactivation-induced cardiac fibrosis by modulating cytokines. J. Cardiovasc. Transl. Res..

[2] Nichtova Z, Novotova M, Kralova E, Stankovicova T (2012). Morphological and functional characteristics of models of experimental myocardial injury induced by isoproterenol. Gen. Physiol. Biophys..

[3] Adamcova M, Baka T, Dolezelova E, Aziriova S, Krajcirovicova K, Karesova I, Stanko P, Repova K, Simko F (2019). Relations between markers of cardiac remodelling and left ventricular collagen in an isoproterenol-induced heart damage model. J. Physiol. Pharmacol..

[4] Ma X, Fu Y, Xiao H, Song Y, Chen R, Shen J, An X, Shen Q, Li Z, Zhang Y (2015). Cardiac fibrosis alleviated by exercise training is AMPK-dependent. PLoS One.

[5] Sozmen M, Devrim AK, Kabak YB, Devrim T, Sudagidan M (2018). The effects of periostin in a rat model of isoproterenol: Mediated cardiotoxicity. Cardiovasc. Toxicol..

[6] Suchal K, Malik S, Gamad N, Malhotra RK, Goyal SN, Bhatia J, Arya DS (2016). Kampeferol protects against oxidative stress and apoptotic damage in experimental model of isoproterenol-induced cardiac toxicity in rats. Phytomedicine.

[7] Tkachenko V, Kovalchuk Y, Bondarenko N, Bondarenko capital OC, Ushakova G, Shevtsova A (2018). The Cardio- and neuroprotective effects of corvitin and 2-oxoglutarate in rats with pituitrin-isoproterenol-induced myocardial damage. Biochem. Res. Int..

[8] Ravindran S, Gopalakrishnan S, Kurian GA (2020). Beneficial effect of sodium thiosulfate extends beyond myocardial tissue in isoproterenol model of infarction: Implication for nootropic effects. J. Biochem. Mol. Toxicol..

[9] Hu Y, Liu X, Zhang T, Chen C, Dong X, Can Y, Liu P (2020). Behavioral and biochemical effects of KXS on postmyocardial infarction depression. Front. Pharmacol..

[10] Pedersen BK, Saltin B (2015). Exercise as medicine—evidence for prescribing exercise as therapy in 26 different chronic diseases. Scand. J. Med. Sci. Sports.

[11] Petersen AM, Petersen BK (2005). The anti-inflammatory effect of exercise. J. Appl. Physiol. (1985).

[12] Chen WW, Zhang X, Huang WJ (2016). Role of neuroinflammation in neurodegenerative diseases (Review). Mol. Med. Rep..

[13] Wexler BC, Greenberg BP (1974). Effect of exercise on myocardial infarction in young vs. old male rats: Electrocardiograph changes. Am. Heart J..

[14] Azamian JA, Hadi A, Haffezi AMR, Javad C (2017). Effect of endurance exercise training on morphological changes in rat heart tissue following experimental myocardial infarction. J. Bas. Res. Med. Sci..

[15] Hovens IB, Schoemaker RG, van der Zee EA, Absalom AR, Heineman E, van Leeuwen BL (2014). Postoperative cognitive dysfunction: Involvement of neuroinflammation and neuronal functioning. Brain Behav. Immun..

[16] Hovens IB, van Leeuwen BL, Mariani MA, Kraneveld AD, Schoemaker RG (2016). Postoperative cognitive dysfunction and neuroinflammation; cardiac surgery and abdominal surgery are not the same. Brain Behav. Immun..

[17] Hovens IB, Nyakas C, Schoemaker RG (2014). A novel method for evaluating microglial activation using ionized calcium-binding adaptor protein-1 staining: Cewll body to cell size ratio. Neuroimmunol. Neuroinflamm..

[18] Engin E, Treit D (2007). The role of hippocampus in anxiety: Intracerebral infusion studies. Behav. Pharmacol..

[19] Wexler BC, Kittinger GW, Judd JT (1967). Responses to drug-induced myocardial necrosis in rats with various degrees of arteriosclerosis. Circ. Res..

[20] Beznak M, Hacker P (1964). Hemodynamics during the chronic stage of myocardial damage caused by isoproterenol. Can. J. Physiol. Pharmacol..

[21] Balakumar P, Singh AP, Singh M (2007). Rodent models of heart failure. J. Pharmacol. Toxicol. Methods.

[22] Heather LC, Catchpole AF, Stuckey DJ, Cole MA, Carr CA, Clarke K (2009). Isoproterenol induces in vivo functional and metabolic abnormalities: Similar to those found in the infarcted rat heart. J. Physiol. Pharmacol..

[23] Woulfe KC, Wilson CE, Nau S, Chau S, Phillips EK, Zang S, Tompkins C, Sucharov CC, Miyamoto SD, Stauffer BL (2018). Acute isoproterenol leads to age-dependent arrhythmogenesis in guinea pigs. Am. J. Physiol. Heart Circ. Physiol..

[24] Nicak A, Machan V, Vilcek S, Kalincak M, Kohut A (1978). Age-dependent variations in the intensity of isoprenaline-induced myocardial lesions in rats. Cor. Vasa.

[25] Shukla SK, Sharma SB, Singh UR (2015). Pre-treatment with alpha-tocopherol and Terminalia arjuna ameliorates, pro-inflammatory cytokines, cardiac and apoptotic markers in myocardial infracted rats. Redox Rep..

[26] Liu H, Luiten PG, Eisel UL, Dejongste MJ, Schoemaker RG (2013). Depression after myocardial infarction: TNF-alpha-induced alterations of the blood-brain barrier and its putative therapeutic implications. Neurosci. Biobehav. Rev..

[27] Quan N (2014). In-depth conversation: Spectrum and kinetics of neuroimmune afferent pathways. Brain Behav. Immun..

[28] Schoemaker RG, Smits JF (1994). Behavioral changes following chronic myocardial infarction in rats. Physiol. Behav..

[29] Svensson M, Lexell J, Deierborg T (2015). Effects of physical exercise on neuroinflammation, neuroplasticity, neurodegeneration, and behavior: What we can learn from animal models in clinical settings. Neurorehabil. Neural Repair.

[30] Brodowicz GR, Lamb DR (1991). Exercise training, indomethacin, and isoproterenol-induced myocardial necrosis in the rat. Basic Res. Cardiol..

[31] Sleiman SF, Henry J, Al-Haddad R, El Hayek L, Abou Haidar E, Stringer T, Ulja D, Karuppagounder SS, Holson EB, Ratan RR, Ninan I, Chao MV (2016). Exercise promotes the expression of brain derived neurotrophic factor (BDNF) through the action of the ketone body beta-hydroxybutyrate. Elife.

[32] Boche D, Perry VH, Nicoll JA (2013). Review: Activation patterns of microglia and their identification in the human brain. Neuropathol. Appl. Neurobiol..

[33] Rinaldi B, Guida F, Furiano A, Donniacuo M, Luongo L, Gritti G, Urbanek K, Messina G, Maione S, Rossi F, de Novellis V (2015). Effect of prolonged moderate exercise on the changes of nonneuronal cells in early myocardial infarction. Neural Plast..

[34] Jensen TE, Richter EA (2012). Regulation of glucose and glycogen metabolism during and after exercise. J. Physiol..

[35] Raefsky SM, Mattson MP (2017). Adaptive responses of neuronal mitochondria to bioenergetic challenges: Roles in neuroplasticity and disease resistance. Free Radic. Biol. Med..

[36] Hirase H, Akther S, Wang X, Oe Y (2019). Glycogen distribution in mouse hippocampus. J. Neurosci. Res..

